# Comparative Genomic Analyses of the Genus *Nesterenkonia* Unravels the Genomic Adaptation to Polar Extreme Environments

**DOI:** 10.3390/microorganisms10020233

**Published:** 2022-01-21

**Authors:** Daoxin Dai, Huibin Lu, Peng Xing, Qinglong Wu

**Affiliations:** 1 State Key Laboratory of Lake Science and Environment, Nanjing Institute of Geography and Limnology, Chinese Academy of Sciences, 73 East Beijing Road, Nanjing 210008, China; daidaoxin2022@163.com (D.D.); luhuibin611@163.com (H.L.); pxing@niglas.ac.cn (P.X.); 2 College of Resources and Environment, University of Chinese Academy of Sciences, Beijing 100049, China; 3 Yunnan Key Laboratory of Plateau Geographical Process and Environmental Changes, School of Tourism and Geography, Yunnan Normal University, Kunming 650500, China; 4 Sino-Danish Center for Science and Education, University of Chinese Academy of Sciences, Beijing 100049, China; 5 The Fuxianhu Station of Deep Lake Research, Chinese Academy of Sciences, Chengjiang 652500, China

**Keywords:** microbial adaptation, comparative genome, polar environments, *Nesterenkonia*

## Abstract

The members of the *Nesterenkonia* genus have been isolated from various habitats, like saline soil, salt lake, sponge-associated and the human gut, some of which are even located in polar areas. To identify their stress resistance mechanisms and draw a genomic profile across this genus, we isolated four *Nesterenkonia* strains from the lakes in the Tibetan Plateau, referred to as the third pole, and compared them with all other 30 high-quality *Nesterenkonia* genomes that are deposited in NCBI. The Heaps’ law model estimated that the pan-genome of this genus is open and the number of core, shell, cloud, and singleton genes were 993 (6.61%), 2782 (18.52%), 4117 (27.40%), and 7132 (47.47%), respectively. Phylogenomic and ANI/AAI analysis indicated that all genomes can be divided into three main clades, named NES-1, NES-2, and NES-3. The strains isolated from lakes in the Tibetan Plateau were clustered with four strains from different sources in the Antarctic and formed a subclade within NES-2, described as NES-AT. Genome features of this subclade, including GC (guanine + cytosine) content, tRNA number, carbon/nitrogen atoms per residue side chain (C/N-ARSC), and amino acid composition, in NES-AT individuals were significantly different from other strains, indicating genomic adaptation to cold, nutrient-limited, osmotic, and ultraviolet conditions in polar areas. Functional analysis revealed the enrichment of specific genes involved in bacteriorhodopsin synthesis, biofilm formation, and more diverse nutrient substance metabolism genes in the NES-AT clade, suggesting potential adaptation strategies for energy metabolism in polar environments. This study provides a comprehensive profile of the genomic features of the *Nesterenkonia* genus and reveals the possible mechanism for the survival of *Nesterenkonia* isolates in polar areas.

## 1. Introduction 

The Qinghai-Tibet Plateau (QTP) is referred to as the third pole of the world because it shares many characteristics with the Arctic and Antarctic regions. The common harsh conditions include low temperature, limited nutrient availability, and strong ultraviolet radiation [1]. In addition, lakes in these regions also suffer from a wide range of salinity and pH, which together contribute to them becoming research hotspots for studies of extremophiles adaption [2,3,4]. Microbial community structures in the Antarctic, Arctic, and Tibetan lakes have been investigated alone and it has been found that the main impact factors include light, temperature, and physicochemical conditions, including the availability of organic carbon and nutrients [5,6,7]. The bacterial diversity and community composition in lakes from the three polar regions were found to also share some common microbial taxa [8]. However, this traditional amplicon sequencing method cannot capture the specific genomic and functional differentiations below the species level. The higher-precision method should be applied to clarify mechanisms by which the bacteria have evolved to survive in these harsh conditions.

Bacterial strains isolated from the polar region have shown many molecular mechanisms for adaptation to extreme conditions. For instance, *Marisediminicola antarctica* ZS314T, isolated from intertidal sediments in East Antarctica, has reddish-orange pigments synthesis capacity at low temperatures [9]. This carotenoid product may contribute to the regulation of membrane fluidity and can also protect cells against UV radiation [10]. Antarctic *Streptomyces* and *Kribbella* strains harbor biosynthetic gene clusters that encodes lipopeptide biosurfactant molecules, the primary ecological role of which is accelerating nutrient flow [11]. The psychrophilic *Arthrobacter* isolate contains more copies of nucleic acid-binding cold-shock proteins (CSPs) and, also, the homologs of the CspA-like cold acclimation protein. These proteins can show different expression patterns to a sudden temperature transition and can help to stabilize DNA and RNA secondary structures during growth under low conditions [12].

*Nesterenkonia*, is a genus within the *Micrococcaceae* family, consisting of many mesophilic moderate haloalkaliphiles from various sources. Members of this group possess high genomic G + C content (64%–72%) and are generally aerobic, gram-positive, and chemo-organotrophic. They have been found in diverse environmental habitats, like hypersaline and saline lakes [13,14,15], cotton pulp mills [16], and the human gut [17,18]. Members belonging to this genus are also isolated from the Antarctic and Arctic [19,20], suggesting that *Nesterenkonia* spp. might have specific adaptation mechanisms to the polar environment. Previous research about the strain *Act20* from the high-altitude-Andean-lake in Argentina found that is has multi-resistance, especially towards UV radiation, drought, and copper [21]. Transcriptional analysis of the Antarctic strain AN1 showed that genes related to antioxidants-coding, cold stress, and the glyoxylate cycle were significantly upregulated during cold growth [20]. However, the systematic analysis of genomic features across all genomes within this genus was absent, which can be resolved by pan-genome analyses. This method can encompass the entire gene pool of target species and offer a framework for genomic diversity estimation [22]. By dividing the full gene repertoire into a few parts (core, shell, cloud, and singleton gene), many important scientific projects can be studied, such as environmental adaptation [23,24], speciation mechanisms [25], and pathogenic drug resistance [26].

In this study, we isolated and sequenced four *Nesterenkonia* strains from different Tibetan lakes and attempted to draw the genomic profile to extreme niche adaptation using the comparative genomic method with all readily published and available genomes of *Nesterenkonia* species in the public National Center for Biotechnology Information (NCBI) database. Among the four strains and other strains from the Arctic and Antarctic regions, we observed some genomic adaptation to cold, nutrient-limited, osmotic, and ultraviolet conditions, as well as energy metabolism adaptation to polar environments.

## 2. Materials and Methods

### 2.1. Sampling, Isolation, and Physiological Measurement

Large-scaled samplings from Tibetan lakes and bacterial isolation were conducted in August of 2015 and 2016. The samples from freshwater and saline lakes were cultivated in Luria–Bertani (LB, 4% NaCl) and CM medium (0.5% NaCl) [27], respectively. Water samples were gradient diluted and plated directly, while the sediment samples were dissolved in the NaCl solution first. The NaCl concentration of the solution was adjusted according to the salinity of its source lake. The taxonomy of the selected colony was confirmed by PCR reaction, which amplified the 16s rRNA with primer pair 27F-1492R. The obtained sequence was blast searching against the EZBioCloud database to find the closest related taxa [28]. Finally, four isolates, which have 99.86% identity with *Nesterenkonia aurantiaca*, were selected to perform further analysis. The strain LB17, AY15, YGD6, and DZ6 are from samples in Lubu Cuo water, Ayong Cuo sediment, Yagedong Cuo water, and Daze Cuo sediment, respectively. The conductivity, pH, water temperature (Temp), and dissolved oxygen (DO) concentration measurements were conducted in the field using a multi-parameter water quality sonde (YSI 6600, Yellow Springs, Greene, OH, USA). Other environment parameters, including total phosphorus, total nitrogen, ammonium, nitrate, nitrite, and phosphate, were analyzed using the standard methods [29].

### 2.2. Whole Genome Sequencing, Assembly, and Reference Genomes Collection

Bacterial genomic DNA was extracted using the SDS method [30]. After evaluating the DNA quality and quantity using NanoDrop 2000 (Thermo Scientific, Waltham, MA, USA), the sequencing libraries were generated using the NEBNext^®^ Ultra™ DNA Library Prep Kit for Illumina (New England Biolabs, MA, USA). Finally, the sequencing process was conducted using Illumina NovaSeq PE150 at the Beijing Novogene Bioinformatics Technology Co., Ltd. The adapter sequence of raw reads was detected using BBmap v37.0 [31] and then removed using Trimmomatic v0.33 [32]. The resulting high-quality reads were assembled to the scaffolds using SPAdes v3.9 [33]. The genome data obtained in this study have been deposited at NCBI under the BioProject number PRJNA786453. The genomes of all *Nesterenkonia* isolates in the Genbank and *Neomicrococcus aestuarii* strain B18 were downloaded using the ncbi-genome-download script (https://github.com/kblin/ncbi-genome-download, accessed on 7 Octobor 2021). In all genomes, only the scaffold with longer than 1000-bp length was kept for downstream analysis.

### 2.3. Genome Quality Estimation, Gene Annotation, and Phylogenetic Analysis

The completeness and contamination of each genome were evaluated using CheckM v1.1.3 [34]. Only the genome with >95% completeness and <5% contamination was selected for downstream analysis. Other genomic statistic parameters were calculated by seqkit v0.16.1 [35]. Gene calling for each genome was performed using PROKKA v1.14.5 [36]. The tRNAscan was used to predict the tRNA [37]. All protein-coding genes were annotated by searching the KEGG KOfam [38] and COGs (Clusters of Orthologous Groups) databases [39] using HMMER v3.3.2 [40]. The 16s rRNA sequences of all genomes were exacted and aligned using mafft v7.474 [41] with the “-auto” command. The maximum likelihood phylogenomic tree was constructed using IQ-TREE v1.6.12 [42] by default parameters. For species-tree building, protein-coding genes were first clustered into orthologous groups (OG) using OrthoFinder v2.2.1 [43]. The sequences in each shared single-copy OG were aligned and trimmed using mafft v7.474 and trimAl v1.2 [44], respectively. The no-gap alignments were concatenated and used as the input to construct a maximum likelihood phylogenomic tree using IQ-TREE v1.6.12.

### 2.4. Comparative Genomic Analysis

The pairwise average nucleotide identity (ANI) analysis of all genomes was performed using FastANI [45]. Amino acid identity (AAI) between each proteome pair was estimated by the online tool AAI-Matrix [46]. The amino acid (AA) composition and average nitrogen and carbon content of each proteome were calculated by a homemade script. All descriptive statistical analysis and difference significance tests were conducted in R (R Core Team, 2020) [47]. Pan-genome analysis and visualization were conducted on the anvi’o platform [48]. Briefly, the pangenome of all 34 *Nesterenkonia* genomes was computed using ‘anvi-pan-genome’ with default –mcl-inflation 2, which uses the Markov Cluster algorithm [49] to cluster the annotating genes into groups. Pan-genome openness was estimated using the ‘micropan’ R package [50] with 100 permutations. The map for pan-genome, core, and accessory gene distribution was performed by the PanGP program with a distance guide (DG) subsampling algorithm [51]. Combined with the KO and COG annotation, clade-specific functions were identified by the ‘anvi-compute-functional-enrichment-in-pan’ command. Only genes with a <0.05 ‘adjusted q-value’, which represents the false-discovery rate adjusted *p*-value corrected for multiple testing, were considered.

## 3. Results and Discussion

### 3.1. Tibetan Isolates Show High Similarities with Antarctic Isolates

**Figure 1:**
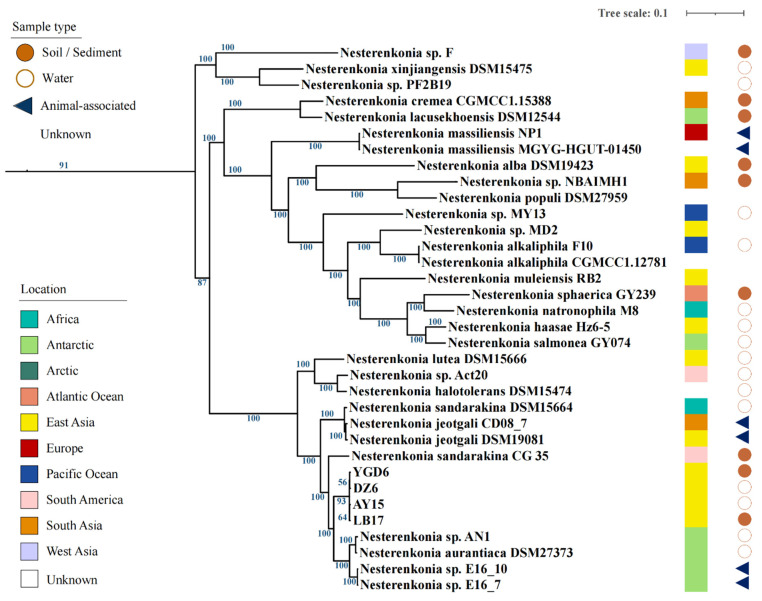
Phylogenetic tree of 34 high-quality *Nesterenkonia* strains. Colored bars and circles/tringles indicate isolation location and habitat type, respectively. The species *Neomicrococcus aestuarii* was used as the outgroup reference genome.

**Figure 2:**
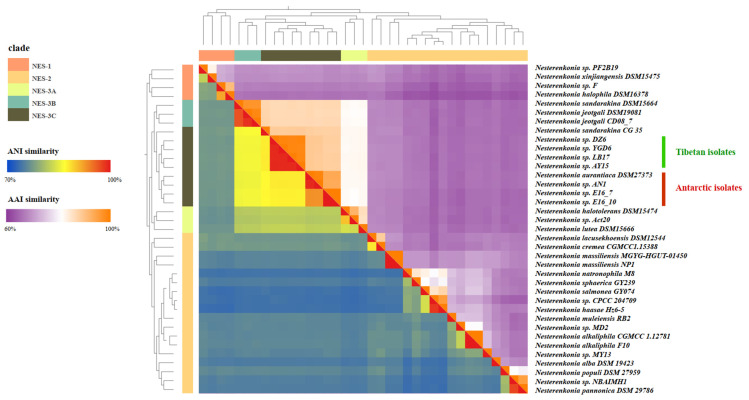
Average nucleotide (ANI, lower left triangle) and amino acid identity (AAI, upper right triangle) analysis of *Nesterenkonia* genus isolates. Genomes were clustered according to the phylogenetic tree.

A total of 6041 orthologous groups common to all *Nesterenkonia* isolates were identified. Phylogenetic analysis based on 304 single-copy OG showed that the whole genus contains three major clades, named herein NES-1, NES-2, and NES-3 (Figure 1, Table 1). The four Tibetan isolates and four Antarctic isolates were found to form a separate subclade (NES-AT) within the major clade NES-2, which may indicate a close evolutionary relationship among these isolates. Phylogenetic analysis based on 16S rRNA gene sequences also indicated the grouping of the eight isolates from the Tibetan and Antarctic regions (Figure S1). The isolates within NES-AT subclade shared 1923 single-copy OGs, which were extracted to build the evolutionary tree. According to the tree topology, Tibetan and Antarctic isolates generate their respective cluster, together with the internal subcluster division, indicating potential local adaptions. For Tibetan clusters, environmental salinity seems to be more important than habitat type for microbial divergence. This is because the strains from saline and freshwater lake samples were clustered separately, whether they were isolated from water or sediment. For the Antarctic cluster, the sponge-associated strain E16_7 and E16_10 as well as the soil-derived strain AN1 and DSM 2737 formed two subclusters respectively, which is consistent with the previous results [52]. ANI and AAI analysis also showed similar results (Figure 2). ANI analysis based on whole-genome comparison showed that NES-AT clade strains shared 93.5% of their identity between them, and below 80% of their identity with all *Nesterenkonia* genomes. These results suggest the evolution of bacteria affiliated with NES-AT clade towards the polar environment. The annual mean temperature in the Tibetan Plateau is lower than 10 °C because of the high elevation (>4000 m) [53], while even in the warmest January, the average air temperature in the Antarctic region was only 2.5 ± 0.49 °C [54]. The cold weather and serious UV radiation in both regions limit the growth of organisms and lead to low productivity [55]. On the other hand, this high similarity can also provide some supports for the hypothesis about the geological history of the two regions. More specifically, Tibet originated from the Gondwanaland plate including India and these eight isolates may come from the common ancestor [56].

### 3.2. Comparative Genomic Analysis

**Figure 3:**
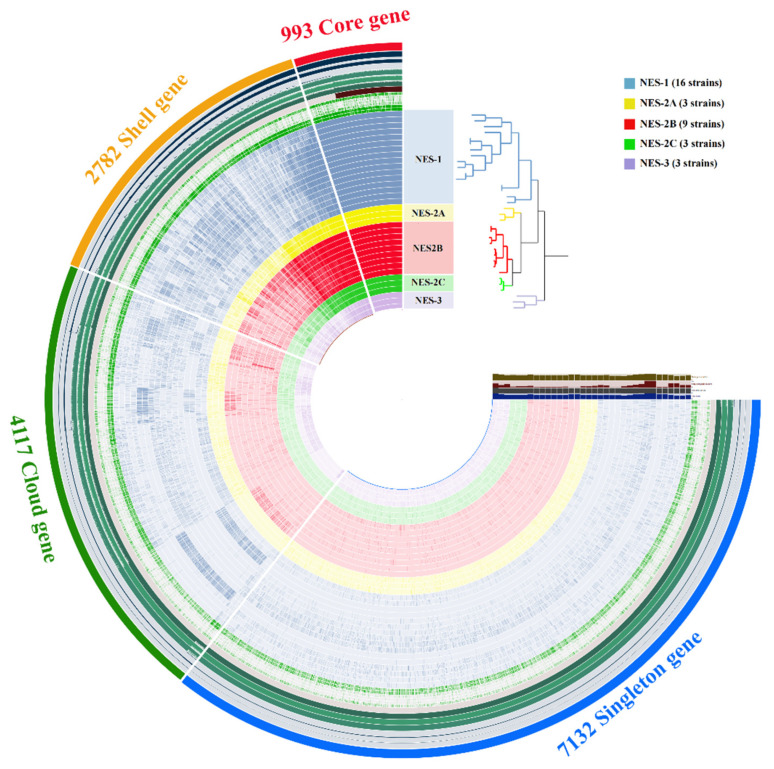
Pan-genome analysis of *Nesterenkonia* genus using Anvi’o workflow. The genomes are organized in radial layers as core, shell, cloud, and singleton gene clusters.

**Figure 4:**
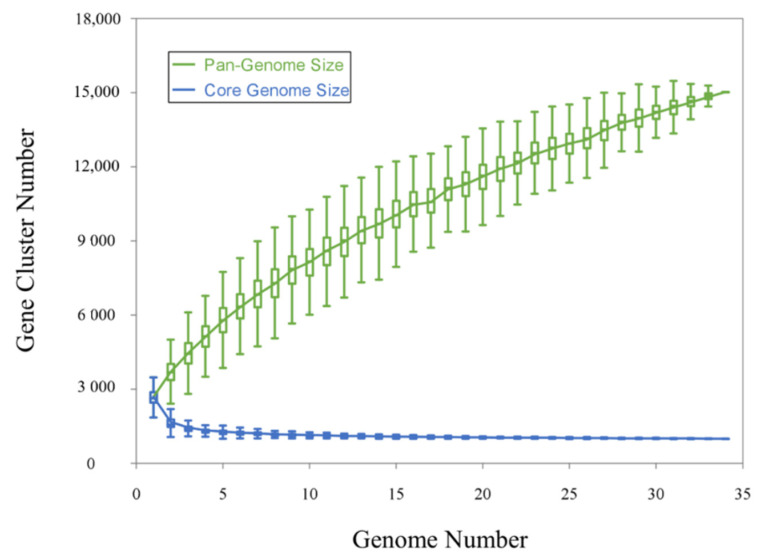
Characteristic curves of the pan-genome and core genome using PanGP.

The pan-genome analysis found a total of 15,024 gene clusters across the 34 high-quality *Nesterenkonia* genomes (Figure 3), which include 30 reference genomes from NCBI and also the strains that we isolated. Gene clusters were defined as core (present in each genome) and accessory (non-core genes) types. The latter was then classified into “shell” (present in 99%-15% genomes), “cloud” (found in less than 15% single genome), and “singleton” (found in only one genome). The number of core, shell, cloud and singleton genes were 993 (6.61%), 2782 (18.52%), 4117 (27.40%), and 7132 (47.47%), respectively. Heaps law model parameter α estimation was equal to 0.3976, less than the threshold of 1.00 [57]. In addition, the gene accumulation curves of the pan-genome show that the power trend line has not arrived at the platform stage (Figure 4). Both results suggest that this genus has an open pan-genome and the sequenced genomes cannot contain the complete gene repertoire. As more *Nesterenkonia* strains are sequenced, more novel genes will be found, leading to a larger pan-genome. This openness indicates that isolates within this genus have great potential to integrate exogenous genetic material and broaden genetic diversity by other evolutionary mechanisms, like recombination and mutation. *Nesterenkonia* strains can thus adapt to diverse habitats by accessory genes acquisition and loss and have a very large and flexible gene pool [58]. 

**Figure 5:**
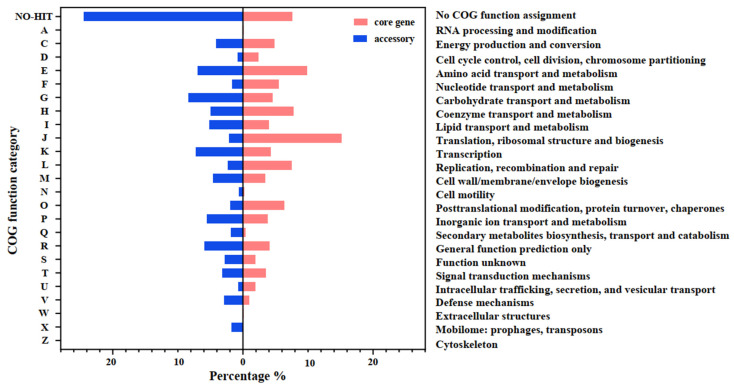
The COG functional categories distribution comparison between core and accessory genes.

We then compared the COG functional categories distribution between the core and accessory genes (Figure 5). As expected, the COG functional categories of some highly conserved and low evolution rate biological processes, such as COG-J (translation, ribosomal structure, and biogenesis), COG-L (replication, recombination, and repair), COG-O (posttranslational modification, protein turnover, chaperones), COG-U (intracellular trafficking, secretion, and vesicular transport), and COG-F (nucleotide transport and metabolism) concentrated more in the core genome but were low in the accessory genes. Instead, COG-V (defense mechanisms), COG-X (mobilome: prophages, transposons), and COG-K (transcription) enrich more in accessory genes, indicating that strains from different sources possess the distinct capacity of genetic material processing [59] and diverse transcription mechanisms to deal with the changing ecological conditions. COG categories corresponding to amino acid (COG-E) and coenzyme (COG-H) transport and metabolism are dominant in the entire genomes but overrepresented in core genes, while the secondary (COG-Q) and carbohydrate metabolism (COG-G) functions have the opposite distribution. This finding is in line with the high secondary metabolites biosynthetic diversity and polysaccharide degradation ability in the Actinobacteria phylum, which are often present in the flexible genome and are varied below the genus level. Finally, genes within COG-S (unknown function), COG-R (general function prediction only) categories, as well as genes without any COG annotation (NO-HIT), were abundant across the pan-genome and had higher proportions in accessory genes.

### 3.3. Genomic Feature Comparison between NES-AT Isolates and Other Nesterenkonia Isolates

**Figure 6:**
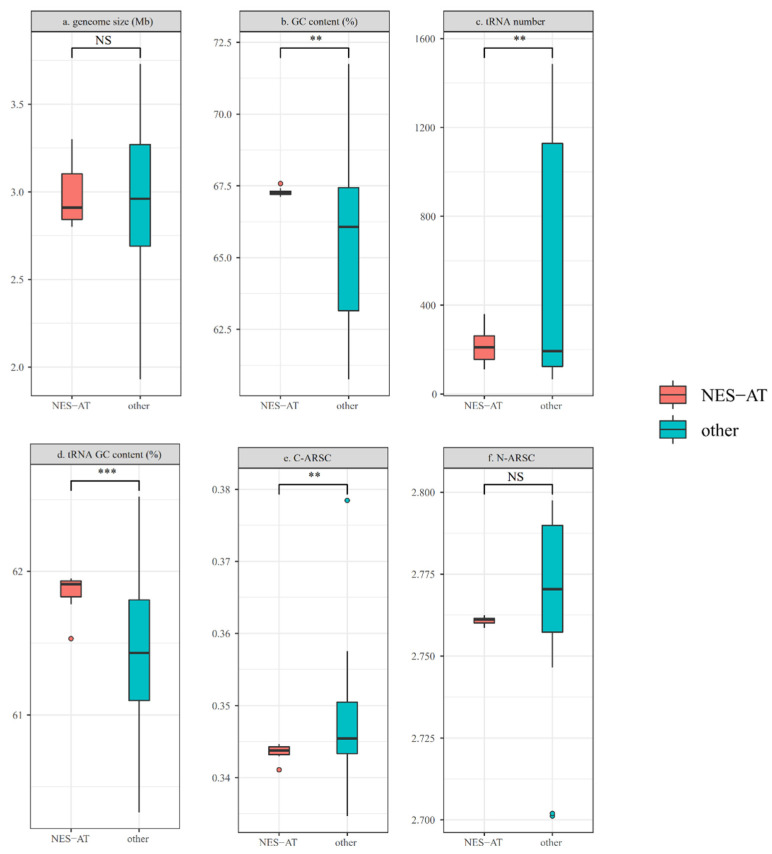
Genomic feature comparisons between NES-AT clades and other references *Nesterenkonia*, marked as NES-AT and other, respectively. (**a**) genome size (Mb), (**b**) GC content (%), (**c**) tRNA number, (**d**) tRNA GC content (%), (**e**) C-ARSC and (**f**) N-ARSC C-ARSC and N-ARSC represent the number of carbon and nitrogen atoms per residue side chain, respectively. (NS: not significant; **: *p* < 0.01; ***: *p* < 0.001). The dot represents the outliers.

Since the bacteria that lived in similar niches shared more genomic features, we calculated some genome characteristic parameters and compared them between NES-AT clade and other isolates (Figure 6).

Genome size (Figure 6a) and GC content (Figure 6b): GC (guanine + cytosine) content, as an important indicator of microbial evolution, is often thought to be positive relative to the genome size [60]. Although the difference in genome size is not significant, the GC content in the NES-AT clade is higher than that in other *Nesterenkonia* strains (*t*-test, *p* < 0.01). Previous studies found that increased GC content often accompanies high rates of genetic damage in the manner of a double-strand break [61], which is often caused by severe ultraviolet radiation [62]. This base composition tendency probably implies the adaptation to serious UV exposure, due to the ozone depletion in the Antarctic and high elevation in Tibetan areas [63,64].

tRNA: Transfer RNAs (tRNA), as an adaptor molecule that participates in the peptide chain synthesis [65], also have an important role in gene expression regulation and cell membrane modification [66]. In addition, yeast can change the tRNA gene abundance (Figure 6c) during stressful conditions, which also reflects its essential function in survival [67]. In *Nesterenkonia* genomes, a higher number of tRNA genes was found in the NES-AT clade (*t*-test, *p* < 0.01, *p* = 0.0067), which showed an opposite tendency in thermophiles [68]. Due to the positive relations between tRNA genes and their relative concentration [69], more tRNA genes may increase the tRNA amount. Higher tRNA numbers can accelerate the transcription/translation speed and make up for the low diffusion rate and metabolic activity in polar environments [70]. Since significant correlations between tRNA abundance and growth rate/optimal growth temperature have been reported in other prokaryotes [68], more tRNA allows organisms to grow fast in the cold. Similar results were also found in other psychrophilic isolates [71]. Mean GC contents of tRNA genes (Figure 6d) for all *Nesterenkonia* genomes were calculated and NES-AT isolates showed significantly higher (*t*-test, *p* < 0.001, *p* = 0.00043) values than others. Similar high tRNA GC% has only been found in hyperthermophiles, the RNA stability of which is needed in high temperatures [72].

C-ARSC (Figure 6e) and N-ARSC (Figure 6f)**:** We also calculated the nitrogen content of protein-coding sequences (N-ARSC) and the numbers of carbon atoms per residue side chain (C-ARSC) in all genomes. These indexes can reflect the nutrient availabilities in the environment, as the comparison studies between epipelagic and mesopelagic *Marinimicrobia* genomic modalities [73]. Only a reduced use of carbon in the AA sequences was found in the NES-AT clade genomes, indicating that carbon-limited conditions in polar regions are likely an important factor influencing the evolution of *Nesterenkonia* [74]. Harsh conditions often limit plant growth, which provides a primary source of organic carbon. Thus, microbes in the polar region usually face the challenge of carbon-poor adaptation [75,76,77].

AA composition: Due to the prevalence of amino acid (AA) preference in microbial cold adaption, AA usage of each proteome was calculated. Since charged polar AA will lead to a stable protein structure by formatting the salt bridge on the protein surface [78], psychrophilic organisms often adjust the AA composition for cold adaption. In NES-AT clade, isolates tend to harbor more nonpolar AA and less polar AA, which includes positively charged histidine, negatively charged aspartate, and glutamine, and uncharged tyrosine and glutamine. The different AA preferences in NES-AT isolates may contribute to the protein flexibility improvement at low temperatures [79]. In addition, the substitution of alanine to glutamine was also found in a psychroactive Antarctic salt-lake archaea *Halorubrum lacusprofundi* [80], which could explain the higher alanine and lower glutamine proportion in NES-AT isolates. Researches about cold-adapted bacterial lipase and cell surface proteins showed remarkably lower aromatic residues [81,82], which is consistent with less tyrosine and tryptophan in the NES-AT clade. Similar results were also found in other prokaryotes groups, like marine *Shewanella* spp. [83] and subzero-growing Arctic permafrost bacteria [84]. A striking feature is a significant leucine preference in the NES-AT clade, which is unfavorable for helical structure flexibility. Leucine, one of the widely used nutrient sources [85], its accumulation might enhance survival in oligotrophic conditions. After all, genome AA composition can also be impacted by environmental concentration [86]. Cystine, another preferred AA that is common to many psychrophiles, is shown in low abundance in NES-AT isolates. Cysteine can form disulfide bonds to assist the cell envelope proteins folding and stability [87]. Reduced content is possibly beneficial to loose protein structure. The third contrary result is shown on phenylalanine, which is nonpolar but capable of cation-π interactions formation [88], whose enrichment is likely to relate with other stress, such as UV defense. This is because phenylalanine is the precursor of mycosporines and mycosporines-like amino acids, which can be used as sunscreen compounds to protect against severe UV damage [89]. Some disagreements with previous studies [90] may arise from species specificity, as there is huge divergence between proteobacteria and actinobacteria phylum.

### 3.4. Functional Genes Related to the “Polar” Environmental Adaption

We first classified all 34 isolates into the “NES-AT” and “Other” clades and compared the COG category difference between them using a student’s *t*-test. We found that the NES-AT clade has significantly more genes in COG-I (lipid transport and metabolism, *p* < 0.001), COG-C (energy production and conversion, *p* < 0.001), COG-B (chromatin structure and dynamics, *p* < 0.001), COG-T (signal transduction mechanisms, *p* < 0.001) and COG-D (cell cycle control, cell division, chromosome partitioning, *p* < 0.01). Lipid is the main component of the cellular membrane and its content and composition can influence membrane fluidity. Bacteria often change the lipid composition of cell walls and membranes for adaptation to cold and oligotrophic conditions. For example, Antarctic *Pseudoalteromonas* isolates PhTAC125 showed better performance in cold adaptation than the closely related strain PspTB41, which contains fewer COG-I genes [91]. COG-C and COG-T classes have been proved to harbor a high number of cold-adapted proteins, which can be helpful for energy acquisition and maintenance under low-temperature stress [92]. Previous transcriptomic analysis of *Nesterenkonia* sp. AN1 in cold response showed that COG-B and COG-D genes were significantly upregulated [20]. This might be consistent with the change of growth cycle and speed when the isolates survive in cold conditions [93]. The same trend is also present in the COG-S category (*p* < 0.01), reflecting the genes with unique and unexplored functions in complex and extreme niches. In contrast, COG-N (Cell motility), COG-*p* (Inorganic ion transport and metabolism, *p* < 0.05), COG-E (*p* < 0.01), and COG-F (*p* < 0.05) related genes have significantly decreased proportions among the NES-AT clade isolates. Oligotrophic bacteria contained fewer genes involved in COG-N because of their lower demand for the transient microscale nutrient sources in the environment [94]. The decreased gene amount of other nutrients (amino acid, nucleotide, and inorganic ion) transporter and metabolism can also be an indication of the adaptive evolution to nutrient-limiting habitats.

Following this, the more specific function enrichment analysis was performed using KOfam and COG annotation results. Some KOfam and COG functions are found to be overrepresented and only the items with an adjusted *q* value < 0.01 were shown (Table 2). Bacteriorhodopsin (COG5524), also called the actinorhodopsin in previous researches, was a putative light-activated proton pump [95]. Its appearance provides NES-AT isolates the potential for the phototrophy lifestyle and improves survival during the nutrient starvation situation, which is similar to the rhodopsin in *Pelagibacter* [96]. Both COG3049 (penicillin V acylase or related amidase from Ntn superfamily) and COG4978 (GyrI-like small molecule binding domain) can act as the transcriptional regulators that control the biofilm formation [97,98], which is a widespread mechanism for bacterial survival under adverse environments. Cellulase/cellobiase (COG5297), most likely the endoglucanase, can give NES-AT isolates the capacity of plant cell wall degradation. Increasing the carbohydrate metabolism diversity can be helpful to energy starvation [99,100]. The glucose they produced can further become the nucleotide sugar precursors (UDP-glucose) and participate in the synthesis of cell surface polysaccharides with the help of O-antigen ligase (COG3307, K16567) [101], which was only enriched in NES-AT isolates. These structures can contribute to diverse biological functions, like nutrient gathering, cold defense, and motility, which protect cells against abiotic and biotic stress [102,103].

NES-AT isolates additionally contain the genes that encoded the NADP-dependent saccharopine dehydrogenase (COG1748, K00290). It can mediate the biosynthesis alpha-aminoadipate pathway of lysine, whose accumulation is a common strategy to block the negative effects of many stress conditions like high salinity [104]. Members of the NES-AT clade also harbor the phosphoenolpyruvate (PEP) synthase (COG0574, K01007) that is capable of catalyzing the PEP to pyruvate with the dependence of AMP and phosphate. As the essential enzyme in glycolysis of the modified Embden-Meyerhof pathway, its appearance would be helpful for energy flux stabilization in energy and ADP-limited environments [105]. More abundant carbon metabolism capacity in the NES-AT clade also reflected on the enrichment of the dimethylamine (DMA) monooxygenase gene cluster (dmmABC, K22342-K22344). This enzyme is required for bacterial growth using DMA, the oxidation product of trimethylamine oxide [106]. On the contrary, the abundance of the gene that encoded the SSS family solute: Na+ symporter (K03307) in other strains is significantly higher than in the NES-AT clade. The solutes carried include many nutrients, like carbohydrates, osmolytes, and cofactors [106]. The above results suggested that in the way of nutrient acquirement, non-polar isolates prefer to absorb from the environments, whereas NES-AT strains tend to broaden the metabolic capacity of alternative carbon substances.

## 4. Conclusions

In this study, four Nesterenkonia strains from the lakes on Tibetan Plateau were isolated and sequenced to identify their stress resistance mechanisms in comparison with all other 30 high-quality Nesterenkonia genomes deposited in the NCBI. The results showed that Tibetan isolates have a close evolutionary relationship with four Antarctic strains and form a subclade NES-AT. Genomes within this clade showed similar genomic properties with other psychrophilic bacteria, such as higher GC content and increased number of tRNA. The reduced use of carbon in the amino acid sequence of NES-AT members is consistent with the nutrient-limited conditions in polar regions. Similar patterns are also present in the results of functional genes enrichment. That is, Tibetan and Antarctic genomes contain more genes that are involved in diverse carbohydrate metabolism and biofilm formation, which can be helpful to stress defense. This study improved our knowledge about how Nesterenkonia strains from Tibetan and Antarctic regions changed their genomic properties and gene content towards adaptation of polar extreme conditions.

## Figures and Tables

**Table 1:** Genomes information of the strains within the genus *Nesterenkonia*.

Strain Name	Accession Number	Scaffold Number	Geneome Size (M)	Gene Number	GC%	rRNA Number	tRNA Number	Completeness	Contamination
*Nesterenkonia* sp. AY15	JAJOYV000000000	20	2,801,805	2574	67.24	5	234	99.01	0.46
*Nesterenkonia* sp. DZ6	JAJOYW000000000	9	2,869,920	2614	67.2	5	360	99.01	0.57
*Nesterenkonia* sp. F	GCA_000220985.2	134	2,809,541	2514	71.49	3	93	98.59	0.52
*Nesterenkonia* alba DSM 19423	GCA_000421745.1	36	2,591,866	2384	63.75	6	140	98.38	0
*Nesterenkonia* massiliensis NP1	GCA_000455245.1	19	2,672,431	2550	62.16	3	261	97.73	0.46
*Nesterenkonia* sp. AN1	GCA_000582475.1	42	3,040,130	2932	67.42	3	167	97.15	0.69
*Nesterenkonia* jeotgali strain CD08_7	GCA_001483765.1	8	2,925,195	2715	67.63	3	364	98.51	0.98
*Nesterenkonia* sp. PF2B19	GCA_001758425.2	134	3,696,919	3701	69.49	5	91	96.95	0.07
*Nesterenkonia* sandarakina strain CG 35	GCA_003003175.1	56	3,224,976	3001	67.46	9	124	99.39	0.46
*Nesterenkonia* natronophila strain M8	GCA_003595215.1	5	2,520,774	2363	61.82	3	402	98.34	0.07
*Nesterenkonia* muleiensis strain RB2	GCA_003600155.1	57	3,676,111	3493	63.55	2	95	97.79	0.86
*Nesterenkonia* salmonea strain GY074	GCA_005771525.1	110	3,267,177	3175	61.13	3	114	99.16	0.34
*Nesterenkonia* sphaerica strain GY239	GCA_005771565.1	52	2,770,794	2633	64.28	4	143	99.03	1.41
*Nesterenkonia* sp. NBAIMH1	GCA_007922635.1	1	2,691,978	2605	66.41	6	1128	97.94	0.18
*Nesterenkonia* populi strain DSM 27959	GCA_007994735.1	2	2,551,278	2414	66.85	6	1177	98.17	0.88
*Nesterenkonia* sp. MD2	GCA_008711175.1	41	3,733,063	3593	63.15	5	142	98.41	1.41
*Nesterenkonia* alkaliphila strain F10	GCA_009758175.1	103	3,318,774	3105	64.83	2	66	98.85	1.43
*Nesterenkonia* haasae strain Hz 6-5	GCA_010119385.1	29	3,422,101	3258	60.8	7	193	99.16	1.21
*Nesterenkonia* sp. MY13	GCA_012641515.1	41	3,101,056	2965	63.07	3	144	98.8	1.68
*Nesterenkonia* sandarakina strain DSM 15664	GCA_013410215.1	2	3,017,448	2780	67.51	6	1128	98.58	1.05
*Nesterenkonia* xinjiangensis strain DSM 15475	GCA_013410745.1	1	3,569,370	3182	68.81	6	1225	99.77	0.61
*Nesterenkonia* jeotgali strain DSM 19081	GCA_014138825.1	1	3,002,985	2767	67.44	6	1275	98.51	3.28
*Nesterenkonia* alkaliphila CGMCC 1	GCA_014639295.1	81	3,386,621	3181	64.79	4	103	98.85	1.43
*Nesterenkonia* cremea CGMCC 1	GCA_014642675.1	37	3,082,200	2850	66.86	5	167	99.56	0.88
*Nesterenkonia* lutea strain DSM 15666	GCA_014873955.1	2	2,958,123	2702	66.73	6	1128	99.54	0.07
*Nesterenkonia* halotolerans strain DSM 15474	GCA_014874065.1	3	2,966,101	2742	66.24	6	648	99.16	1.28
*Nesterenkonia* sp. E16_7	GCA_017347075.1	82	3,294,162	3074	67.28	3	111	98.88	1.44
*Nesterenkonia* sp. E16_10	GCA_017347085.1	49	3,295,232	3071	67.28	3	122	98.42	1.44
*Nesterenkonia* lacusekhoensis strain DSM 12544	GCA_017876395.1	2	2,742,649	2662	66.68	6	1486	100	0.99
*Nesterenkonia* sp. Act20	GCA_019173455.1	2	2,930,097	2732	65.93	7	697	99.58	0.75
*Nesterenkonia* massiliensis MGYG-HGUT-01450	GCA_902375145.1	19	2,672,431	2550	62.16	3	261	97.73	0.46
*Nesterenkonia* sp. LB17	JAJOYX000000000	10	2,819,602	2569	67.19	4	297	99.01	0
*Nesterenkonia* aurantiaca strain DSM 27373	GCA_004364585.1	25	2,948,026	2704	67.58	4	186	99.11	0
*Nesterenkonia* sp. YGD6	JAJOYY000000000	11	2,853,887	2592	67.12	3	250	99.01	0

**Table 2:** KOfam and COG functions enrichment summary.

**Function Class**	**COG Function**	**Enrichment Score**	**Adjusted *q* Value**	**Enriched Groups**	**Accession**
Energy production	Bacteriorhodopsin	37	0	AT	COG5524
Transcriptional regulators	Penicillin V acylase or related amidase, Ntn superfamily (YxeI)	37	0	AT	COG3049
	GyrI-like small molecule binding domain (BltR2)	31.7525	0	AT	COG4978
	HD superfamily phosphodieaserase, includes HD domain of RNase Y (RnaY)	16.3079	0.0061	AT	COG1418
Polysaccharides metabolism	Cellulase/cellobiase CelA1 (CelA1)	27.5549	0.0001	AT	COG5297
	O-antigen ligase (RfaL)	24.1298	0.0004	AT	COG3307
	Phosphoglycerol transferase MdoB/OpgB, AlkP superfamily (MdoB)	14.9693	0.0089	AT	COG1368
Glycolysis	Phosphoenolpyruvate synthase/pyruvate phosphate dikinase (PpsA)	18.8444	0.0024	AT	COG0574
Lysine metabolism	Saccharopine dehydrogenase, NADP-dependent (Lys9)	18.8444	0.0024	AT	COG1748
Ion transporters	H^+^/Cl^−^ antiporter ClcA (ClcA)	18.8444	0.0024	AT	COG0038
	Mg^2+^ and Co^2+^ transporter CorA (CorA)	14.9693	0.0089	AT	COG0598
Electron transfer chain	Flavodoxin (FldA)	18.8444	0.0024	AT	COG0716
	Flavodoxin/ferredoxin-NADP reductase (Fpr)	18.8444	0.0024	AT	COG1018
	Fe-S cluster carrier ATPase, Mrp/ApbC/NBP35 family (Mrp)	14.9693	0.0089	AT	COG0489
Cell motility	Flagellar motor protein MotB (MotB)	18.8444	0.0024	other	COG1360
Cell surface structure	Sialic acid synthase SpsE, contains C-terminal SAF domain (SpsE)	14.9693	0.0089	other	COG2089
	CDP-glycerol glycerophosphotransferase, TagB/SpsB family	14.9693	0.0089	other	COG1887
	Murein tripeptide amidase MpaA (MpaA)	17.1582	0.0054	AT	COG2866
	Thiol:disulfide interchange protein DsbD (DsbD)	16.7683	0.0058	AT	COG4232
Unknown	Uncharacterized conserved protein YchJ, contains N- and C-terminal SEC-C domains (YchJ)	14.9693	0.0089	AT	COG3012
	Uncharacterized membrane protein YccF, DUF307 family (YccF)	14.9693	0.0089	AT	COG3304
	Predicted peptidase	16.7683	0.0058	AT	COG4099
**Function Class**	**KOfam**	**Enrichment Score**	**Adjusted *q* Value**	**Enriched Groups**	**Accession**
Polysaccharides metabolism	exopolysaccharide production protein ExoQ	31.7525	0	AT	K16567
Lysine metabolism	saccharopine dehydrogenase (NAD+, L-lysine forming)	31.7525	0	AT	K00290
Glycolysis	pyruvate, water dikinase	21.2667	0.0012	AT	K01007
Solute transporter	solute: Na+ symporter, SSS family	24.1298	0.0006	other	K03307
	ethanolamine permease	14.9693	0.0095	AT	K16238
	putative amide transporter protein	18.8444	0.0029	AT	K22112
Dimethylamine oxidation	dimethylamine monooxygenase subunit B	21.2667	0.0012	AT	K22343
	dimethylamine monooxygenase subunit C	21.2667	0.0012	AT	K22344
	dimethylamine monooxygenase subunit A	21.2667	0.0012	AT	K22342
Cell surface structure	prokaryotic ubiquitin-like protein Pup	20.925	0.0012	other	K13570
	3-deoxy-manno-octulosonate cytidylyltransferase (CMP-KDO synthetase)	14.9693	0.0095	other	K00979
	N5-(carboxyethyl)ornithine synthase	14.9693	0.0095	AT	K00298
	phosphoglycerol transferase	14.9693	0.0095	AT	K01002
Stress defense	glyoxylase I family protein	18.8444	0.0029	AT	K08234
Rhamnose metabolism	rhamnulokinase	16.7683	0.0073	other	K00848
Methionine biosynthesis	5-methyltetrahydropteroyltriglutamate-homocysteinmethyltransferase	16.7683	0.0073	other	K00549
Methanogenesis	formylmethanofuran dehydrogenase subunit E	16.2576	0.0088	AT	K11261
Antibiotic resistance	fluoroquinolone resistance protein	14.9693	0.0095	AT	K18555
Transcriptional regulators	MarR family transcriptional regulator, lower aerobic nicotinate degradation pathway regulator	14.9693	0.0095	AT	K22296
Lipid metabolism	mitochondrial enoyl-[acyl-carrier protein] reductase/trans-2-enoyl-CoA reductase	14.9693	0.0095	AT	K07512
	4′-phosphopantetheinyl transferase	14.9693	0.0095	AT	K06133
	sterol 3beta-glucosyltransferase	14.9693	0.0095	AT	K05841
Unknown	SEC-C motif domain protein	14.9693	0.0095	AT	K09858

## Data Availability

The genomes of four isolates (sp. AY15, DZ6, LB17 and YGD6) in this study were submitted to the GenBank database under accession numbers JAJOYV000000000, JAJOYW000000000, JAJOYX000000000 and JAJOYY0000000001, respectively.

## Supplementary Materials

The following supporting information can be downloaded at: https://www.mdpi.com/article/10.3390/microorganisms10020233/s1, Figure S1: Phylogenetic tree based on 16S rRNA gene sequences of genus Nesterenkonia.
