# Supplementary material for: Comparative Genomic Analyses of the Genus Nesterenkonia Unravels the Genomic Adaptation to Polar Extreme Environments
Source: Microorganisms. 2022 Jan 21;10(2):233. doi: 10.3390/microorganisms10020233 (PMC8875376; doi:10.3390/microorganisms10020233)
Supplement: Supplementary file 1 [file microorganisms-10-00233-s001.zip › microorganisms-1535599-supplementary.pdf]

microorganisms

MDPI

Comparative Genomic Analyses of the Genus *Nesterenkonia* Unravels the Genomic Adaptation to Polar Extreme Environments

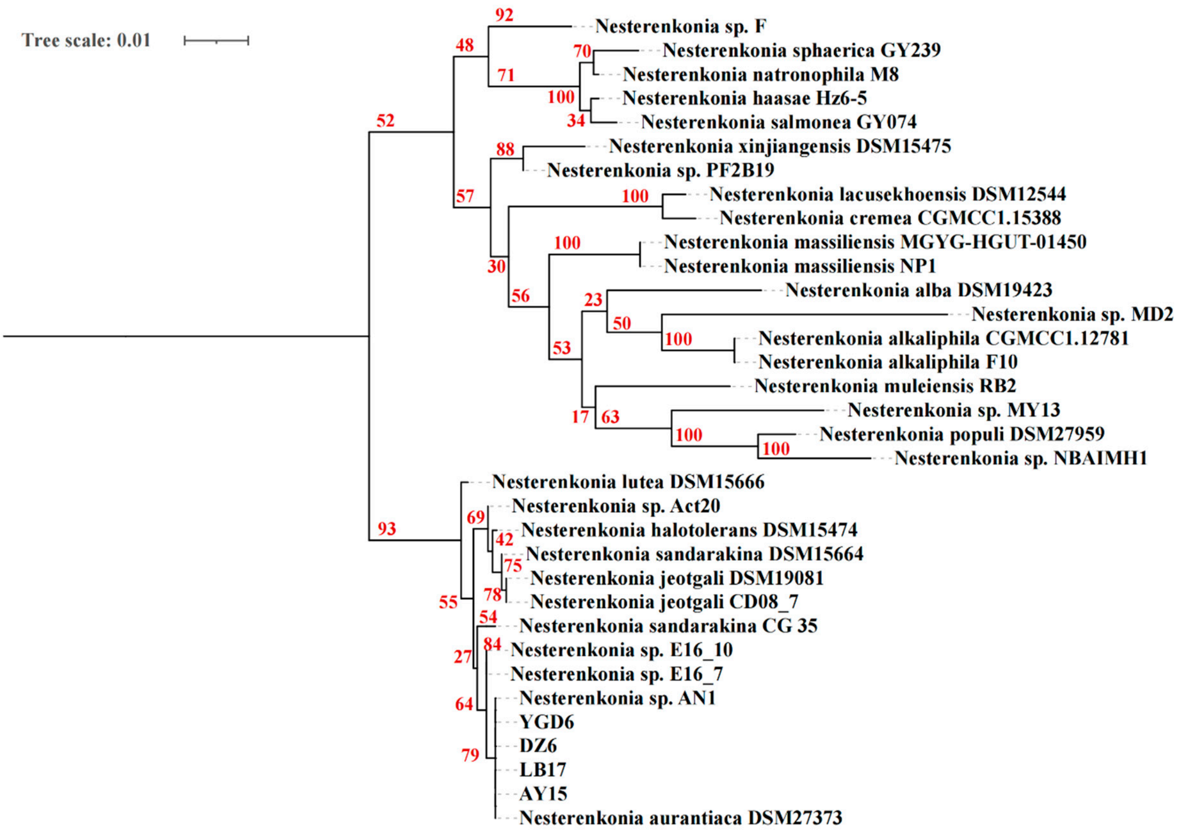

Figure S1. Phylogenetic tree based on 16S rRNA gene sequences of genus *Nesterenkonia*. The four isolated strains from lakes in the Tibetan Plateau were referred as DZ6, YGD6, LB17, and AY15 in this phylogenetic tree. The four strains isolated from the Tibetan Plateau and four strains isolated from Antarctica were grouped together forming a deep branch within this tree.
